# Effect of Ibandronate Therapy on Serum Chemerin, Vaspin, Omentin-1 and Osteoprotegerin (OPG) in Postmenopausal Osteoporotic Females

**DOI:** 10.3389/fphar.2022.822671

**Published:** 2022-02-09

**Authors:** Saba Tariq, Sundus Tariq, Shahad Abduljalil Abualhamael, Muhammad Shahzad

**Affiliations:** ^1^ Department of Pharmacology and Therapeutics, University Medical and Dental College, The University of Faisalabad, Faisalabad, Pakistan; ^2^ Department of Pharmacology, University of Health Sciences, Lahore, Pakistan; ^3^ Department of Physiology and Cell Biology, University Medical and Dental College, The University of Faisalabad, Faisalabad, Pakistan; ^4^ Department of Physiology, University of Health Sciences, Lahore, Pakistan; ^5^ Department of Internal Medicine, King Abdulaziz University, Jeddah, Saudi Arabia

**Keywords:** ibandronate, chemerin, vaspin, omentin-1, OPG, osteoporosis

## Abstract

Osteoporosis is a condition in which bone mineral density is reduced due to altered bone microstructure, which results in increased skeletal fragility and incidence of various types of fractures. Adipokines such as chemerin, vaspin, omentin-1 and osteoprotegerin are involved in bone remodeling. The current study was designed to determine the changes in circulating chemerin, vaspin, omentin-1, and osteoprotegerin levels after treatment with oral ibandronate 150 mg in postmenopausal osteoporotic females. The present study enrolled 107 postmenopausal osteoporotic females from a tertiary care hospital in Faisalabad, Pakistan, based on stringent inclusion and exclusion criteria. Sixty-six healthy postmenopausal, non-osteoporotic females with no systemic illness were chosen from the general population. The assessment of bone mineral density (BMD) was done using a DEXA scan. Serum levels of chemerin, vaspin, omentin-1 and osteoprotegerin were estimated using commercially available enzyme-linked immunosorbent assay kits. The collected data were analyzed with the Statistical Package for Social Sciences (SPSS) version 24. Following 6 months of ibandronate treatment, there was a significant decrease of 24.24% (*p* < .033) in serum chemerin levels, as well as a significant increase in serum vaspin levels 343.32% (*p* < .001) and osteoprotegerin levels 19.57% (*p* < .001), with no significant change in omentin-1 levels. Thus, an increase in serum chemerin levels and a decrease in serum vaspin and osteoprotegerin levels could be implicated in osteoporosis.

## Introduction

Osteoporosis is a chronic bone disease marked by a decreased bone mineral density (BMD) because of altered bone microstructure, leading to increased skeletal fragility and fracture risk (Falaschi and Giordano, 2017). In Pakistan, numerous factors like illiteracy, financial instability, and sedentary lifestyle increase osteoporosis risk among women. The diagnosis modalities such as DEXA scan are not easily accessible, and treatment is expensive (Tariq et al., 2017).

Novel adipokines, including leptin, chemerin, omentin, vaspin, and visfatin, have many physiological functions in the body, and now the researchers are greatly interested in exploring the relationship between these adipokines and bone homeostasis (Liu et al., 2013). Chemerin, a versatile novel adipokine, is a 14-kDa protein secreted by adipose tissue and liver (Menzel et al., 2016). Chemerin plays a major role in different human pathophysiological processes (Fatima et al., 2014). A mice study observed chemerin involvement in bone loss, and the administration of chemerin receptor antagonist CCX832 resulted in inhibition of bone loss. It showed chemerin could increase osteoclastic activity (Ramos-Junior et al., 2017). In contrast to this study, a recent study discovered that CMKLR1 knockout mice have lower trabecular bone mass (Zhao et al., 2019).

Vaspin, a serine protease inhibitor, is derived from visceral adipose tissue and is also known as SERPINA12 according to serpin nomenclature (Weiner et al., 2018). *In vitro*, the apoptosis of human osteoblasts was prevented by vaspin (Zhu et al., 2013). In one of the studies conducted in ambulatory postmenopausal females’ serum vaspin level was positively correlated with BMD at the femoral neck after adjusting various baseline characteristics (Tanna et al., 2017). In addition to this study, another study investigated different inflammatory cytokines and observed that serum vaspin levels were lower in the diabetic group with osteoporosis than the diabetic group without osteoporosis (Li et al., 2016). Low levels of vaspin are involved in the advancement of diabetic osteoporosis through different ways that impact bone metabolism (Li et al., 2016).

Omentin-1 is a novel adipokine consisting of 313 amino acids and is secreted from visceral adipose tissue (Watanabe et al., 2017). The role of omentin-1 in bone homeostasis is also controversial. It has been proposed that omentin-1 increases osteoblast proliferation (Wu et al., 2013) and it also is capable of decreasing the formation of osteoclast by increasing levels of osteoprotegerin in mouse osteoblasts (Xie et al., 2012).

OPG, a protein secreted by osteoblasts, has the ability to bind with the Receptor Activator of Nuclear Factor Kappa-Β Ligand (RANKL) and is a natural inhibitor of RANKL. In premenopausal females, this process is balanced; however, in postmenopausal females decrease in estrogen leads to increase expression of RANKL. It then bypasses OPG and leads to increase binding with RANK causing an increase in osteoclast function and increase in bone resorption, which eventually leads to osteoporosis (Park et al., 2017). The role of OPG as a biomarker in patients with osteoporosis is under consideration.

Oral nitrogen-containing bisphosphonates (e.g., ibandronate) are the standard of care in osteoporosis (Rosen et al., 2017). Randomized clinical studies have shown their effectiveness in the management of osteoporosis, especially in postmenopausal women. In a study conducted in postmenopausal osteoporotic females ibandronate significantly and safely increased BMD throughout the course of 30 months of treatment (Uehara et al., 2021). Similarly, in another study oral ibandronate 100 mg showed equivalent BMD increases to monthly intravenous ibandronate, indicating that it has a high value in the lifestyle and disease factors associated with osteoporosis (Hagino et al., 2018).

A review of the literature indicated no consensus on the roles of chemerin, vaspin, omentin-1, and OPG in bone metabolism. The current study was designed to determine the changes in circulating chemerin, vaspin, omentin-1, and osteoprotegerin levels after treatment with oral ibandronate 150 mg in postmenopausal osteoporotic females.

## Materials and Methods

This experimental study was conducted for 2 years (2018–2020), after taking ethical approval from the Institutional Review Board of the University of Health Sciences, Lahore, Pakistan. Postmenopausal females were recruited for this study during routine visits to the Orthopaedic clinic and OPD at Madina Teaching Hospital in Faisalabad. A sample size of 37 was calculated using the following formula with 90 percent power of the study and 95 percent confidence level, but it was increased to 107 to validate study results.

$${\text{n}=2\text{S}}^{2}{\left({\text{Z}}_{\alpha }{+\text{Z}}_{\beta }\right)}^{2}/\;d^{2}$$

Where α(two-tailed)= 0.050 , β=0.100 , E =0.900 , S(Δ)=1.240

The normal deviation from the standard for α=Zα =1.960

The normal deviation from the standard forβ= Zβ=1.282

$$\text{A }=\;1\text{.}000\text{, B }=\;{\left({\text{Z}}_{\alpha }{+\text{Z}}_{\beta }\right)}^{2}=10\text{.}507\text{, C}={\left(\text{E/}\text{S}\left(\Delta \right)\right)}^{2}=\text{0}\text{.}592\text{,}\text{AB}\text{/}\text{C}$$

Purposive non-randomized sampling was used, and all postmenopausal females aged 50–70 years with amenorrhea for more than 2 years were included in this study. Females with any history of ischemic heart diseases, diabetes mellitus, epilepsy, malignancies, osteomalacia, thyroid, parathyroid and gastrointestinal disease were excluded from the study. Similarly, females with a history of corticosteroids use or drugs to prevent BMD loss such as bisphosphonates, denosumab, calcitonin, PTH, estrogen therapy, androgens, and SERM’s were also excluded from the study. If the subject was taking vitamin D or calcium supplements, a 4 week washout period was implemented (Mann et al., 2014).

### Estimation of Bone Mineral Density

Initially, the BMD of 2,800 females was measured on the calcaneus with a peripheral ultrasound bone densitometer (heel). Subjects diagnosed as osteoporotic using calcaneal ultrasonography were sent for a DEXA scan to begin treatment. BMD of postmenopausal females was measured using a HOLOGIC-HORIZON-A (QDR-series) version 5.6.0.4, Dual-Energy X-ray Absorptiometry (DXA) system at the Pakistan Institute of Nuclear Medicine (PINUM) Hospital, Faisalabad, Pakistan. DEXA estimates the areal BMD in g/cm^2^ that quantifies the skeletal status.

One hundred seventy-three patients were chosen to participate in the study after assessment with DEXA and using strict inclusion and exclusion criteria. All study participants signed a written informed consent form. These 173 patients were divided into two groups. The first group consisted of 66 healthy control postmenopausal females, while the second group consisted of 107 postmenopausal newly diagnosed osteoporotic patients. All subjects were thoroughly examined, and detailed histories were taken and recorded on a specially designed proforma. All subjects’ height, weight, waist and hip circumferences were measured.

As a control, 66 healthy age and sex-matched postmenopausal females were used to investigate normative values of chemerin, vaspin, omentin-1, and OPG in the Pakistani population, as these values had not been studied previously. These females had no endocrine and systemic illness. Blood samples were taken only once in the control group to compare their baseline parameters with those of the patients (Zojer et al., 2005; Martini et al., 2007).

One hundred and seven postmenopausal osteoporotic women with T scores less than or equal to –2.5 were included in the treatment group. Their blood was drawn at the start of the study and again after 6 months of bisphosphonate treatment to determine the effect of bisphosphonate therapy on serum chemerin, vaspin, omentin-1, and OPG levels. Each patient received one bisphosphonate tablet (Ibandronate 150 mg) once a month for a total of 6 months.

### Biochemical Analysis

Ten mL of fasting blood was collected from an antecubital vein using an aseptic technique. Centrifugation was used to separate the serum, which was then stored at −70°C until it was analyzed. Baseline investigations were done to recruit the patients according to inclusion and exclusion criteria. These investigations included complete blood count (CBC), liver function tests (LFTs), renal function tests (RFTs), serum calcium and phosphate levels.

The serum was analyzed for chemerin, vaspin, omentin-1, and OPG first at baseline and again after 6 months of ibandronate treatment using Enzyme-Linked Immunosorbent Assay (ELISA), provided by Elabscience Biotechnology United States. The assay was performed using microplate data collection and analysis software Gen5™ and Gen5 Secure, manufactured by BioTek^®^ Instruments, Inc. According to the manufacturer, the sensitivity of serum chemerin, vaspin, omentin-1, and OPG assays was 0.10, 37.50, 0.38, and 0.10 ng/ml, respectively, whereas the coefficient of variation for serum chemerin, vaspin, omentin-1, and OPG was 10% and cross-reactivity was almost nil. The blood sugar levels were determined using a glucose assay kit.

### Statistical Analysis

For statistical analysis, the Statistical Package for Social Sciences (SPSS) version 24.0 was used. Shapiro-Wilk statistics tested the distribution of data, and if p was <.05, the data was considered non-normally distributed. Median IQR (interquartile range) was given for non-normally distributed quantitative variables. For categorical variables, frequencies and percentages were given. Proportions and percentages were compared using the Chi-square test. Spearmon’s Rho correlation coefficients were used to test the relationship of BMD with serum chemerin, vaspin, omentin-1, and OPG. The independent sample *t*-test and Mann-Whitney U tests were used to compare normal and osteoporotic groups for normally and non-normally distributed data. The Wilcoxon signed-rank test was used to compare pre and post-treatment cases.

## Results

The median age of the study population was 56.50 (52.5-64) and 57 (54-65) years in the control and osteoporotic groups, respectively. Table 1 compares the general characteristics and biochemical parameters of the study groups. Only the number of years since menopause (*p* < .001), weight (*p* < .001), BMI (*p* < .001), and BMD (*p* < .001) differed significantly between the two groups. Serum chemerin levels were significantly higher (*p* < .001) in the osteoporotic group, whereas serum OPG levels were significantly lower (*p* < .001). There was no significant difference in serum vaspin and omentin-1 levels (Table 1).

**TABLE 1:** General characteristics and biochemical parameters of the study population.

Parameters	Control *n* = 66	Osteoporotic *n* = 107	*p*-value
Age of patient (years)	56.50 (52.5–64)	57 (54–65)	.081
Years since menopause	5 (Liu et al., 2013; Zhu et al., 2013; Fatima et al., 2014; Menzel et al., 2016; Ramos-Junior et al., 2017; Tanna et al., 2017; Tariq et al., 2017; Weiner et al., 2018; Zhao et al., 2019)	8 (Liu et al., 2013; Zhu et al., 2013; Fatima et al., 2014; Li et al., 2016; Menzel et al., 2016; Ramos-Junior et al., 2017; Tanna et al., 2017; Watanabe et al., 2017; Weiner et al., 2018; Zhao et al., 2019)	.001*
Height (m)	1.55 (1.52–1.57)	1.55 (1.50–1.57)	.066
Weight (kg)	75.15 (68–85.25)	60 (50–70)	<.001*
BMI (kg/m^2^)	31.98 (27.38–35.36)	25.87 (22.30–29.03)	<.001*
Waist to hip ratio	0.97 (0.94–1.03)	1.00 (0.95–1.04)	.246
Haemoglobin g/dl	12.80 (11.60–13.80)	12.80 (12.10–13.50)	.391
Red Blood Cells mil/mm^3^	5.01 (4.63–5.40)	4.79 (4.52–5.05)	.012
White Blood Cells mm^3^	8.20 (7.28–9.72)	8.70 (7.40–10.20)	.155
Platelets mm^3^	285.50 (253–355.5)	284 (234–331)	.205
Alkaline phosphatase U/L	93 (77–112.25)	102 (80–123)	.137
Alanine transaminase U/L	23 (18–30.25)	20 (Zojer et al., 2005; Martini et al., 2007; Kanaya and Chen, 2010; Muruganandan et al., 2010; Tohidi et al., 2012; Xie et al., 2012; Kamio et al., 2013; Muruganandan et al., 2013; Mann et al., 2014; Makrilakis et al., 2015; Engin-Üstün et al., 2016; Neumann et al., 2016; Terzoudis et al., 2016; Park et al., 2017; Rosen et al., 2017; Dikker et al., 2018; Hagino et al., 2018; Kadric et al., 2018; Rana Ali Hamdi, 2018; Rao et al., 2018; Zhu et al., 2019; Uehara et al., 2021)	.144
Creatinine mg/dL	0.60 (0.50–0.70)	0.60 (0.60–0.80)	.090
Calcium mg/dL	9.65 (9.38–9.90)	9.50 (9.20–9.90)	.220
Phosphate mg/dL	3.80 (3.40–4.10)	3.90 (3.50–4.20)	.214
Lumbar	0.20 (−0.50–1.00)	−2.70 (−3.30 to −2.50)	<.001*
Right Femoral Neck	0.20 (−0.33–0.92)	−2.30 (−2.70 to −1.60)	<.001*
Right Hip	0.35 (−0.23–1.05)	−1.90 (−2.50 to −1.20)	<.001*
Left Femoral Neck	0.10 (−0.60–0.73)	−2.10 (−2.60 to −1.40)	<.001*
Left Hip	0.45 (−0.10–1.00)	−1.70 (−2.20 to −1.00)	<.001*
Total BMD	0.28 (−1.75 to 0.77)	−2.1 (−2.5 to −1.8)	<.001*
Chemerin ng/mL	0.137 (0.083–0.279)	0.231 (0.138–0.508)	<.001*
Vaspin ng/mL	0.686 (0.191–1.561)	0.561 (0.163–1.454)	.400
Omentin-1 ng/ml	7.293 (2.094–14.239)	3.885 (1.773–10.660)	.060
OPG ng/mL	13.185 (11.476–14.638)	11.469 (9.676–13.043)	<.001*

* *p* ≤ .05 is considered statistically significant.

Values are given as Median (IQR).

**FIGURE 1:**
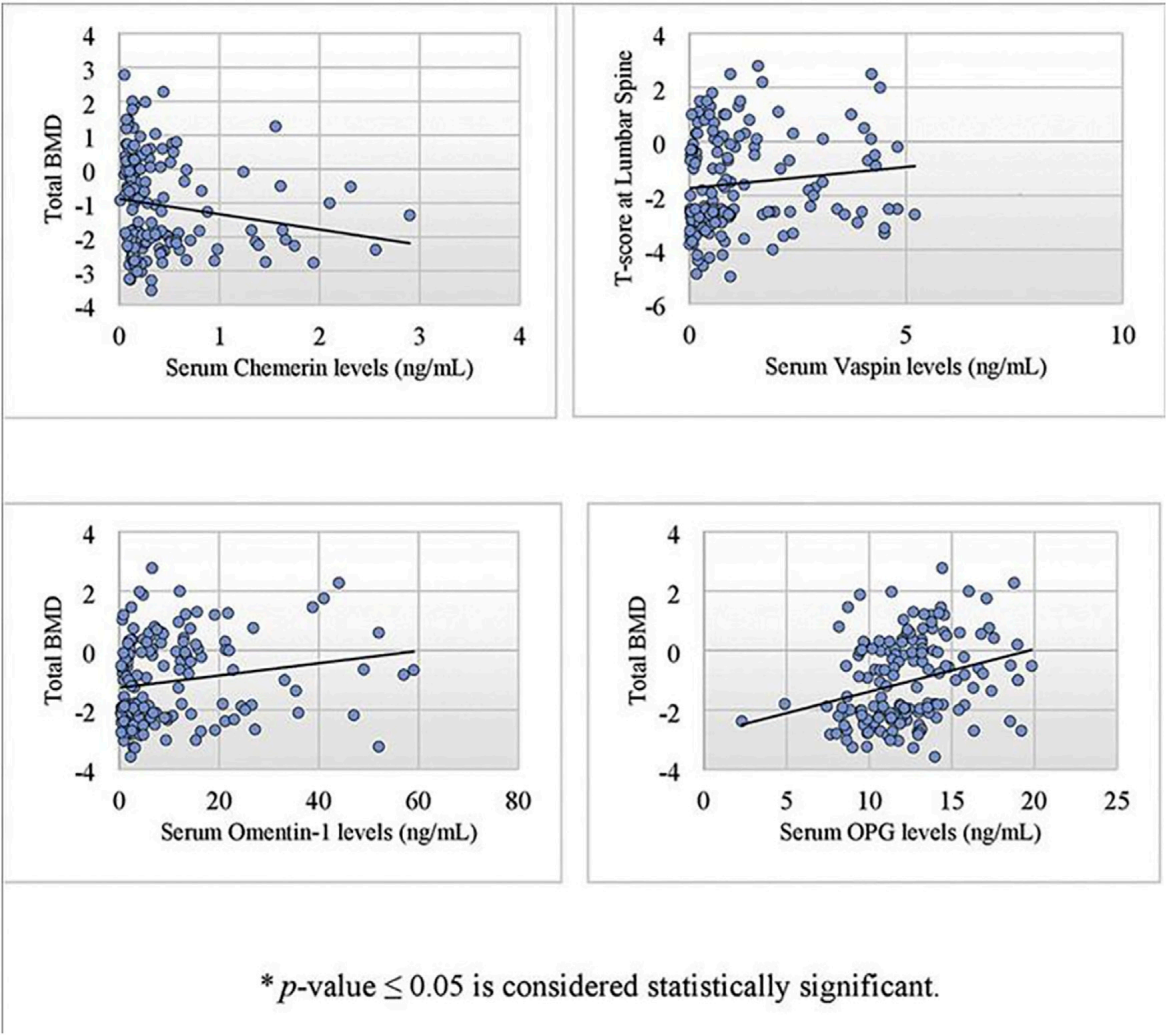
Scatter plot showing a significant correlation of serum chemerin, vaspin, omentin-1 and OPG with bone mineral density in postmenopausal females using spearman’s rho correlation coefficient.

Correlation of biochemical parameters and BMD in postmenopausal females (*n* = 173) is given in Table 2. Serum chemerin had a significant negative correlation with BMD at the lumbar spine (r = −0.217, *p* = .004*), right femoral neck (r = −0.217, *p* = .004*), right hip (r = −0.192, *p* = .011*), left femoral neck (r = −0.246, *p* = .001*), left hip (r = −0.189, *p* = .013*) and total BMD (r = −0.238, *p* = .002*). Serum vaspin was found to have a significant positive correlation with omentin-1 (r = 0.785, *p* = .001*) and BMD at the lumbar spine (r = 0.173, *p* = .023*). Serum omentin-1 was found to have a significant positive correlation with BMD at the lumbar spine (r = 0.232, *p* = .002*), right femoral neck (r = 0.181, *p* = .017*), right hip (r = 0.179, *p* = .018*), left femoral neck (r = 0.178, *p* = .019*), left hip (r = 0.225, *p* = .003*), and total BMD (r = 0.215, *p* = .005*). Serum OPG had a significant positive correlation with BMD at the lumbar spine (r = 0.247, *p* = .001*), right femoral neck (r = 0.302, *p* = .001*), right hip (r = 0.236, *p* = .002*), left femoral neck (r = 0.233, *p* = .002*), left hip (r = 0.280, *p* = .001*), and total BMD (r = 0.295, *p* < .001*) (Figure 1).

**TABLE 2:** Correlation between serum chemerin, vaspin, omentin-1, OPG and BMD in postmenopausal females (*n* = 173).

Parameters Rho	Chemerin Rho	*p-*value*	Vaspin rho	*p*-value*	Omentin-1	*p*-value*	OPG rho	*p*-value*
Chemerin	—	—	0.040	.599	-0.066	.389	0.110	.151
Vaspin	0.040	.599	—	—	0.785	<.001*	0.148	.053
Omentin-1	−0.066	.389	0.785	<.001*	—	—	0.136	.075
OPG	0.110	.151	0.148	.053	0.136	.075	—	—
Lumbar spine BMD	−0.217	.004*	0.173	.023*	0.232	.002*	0.247	.001*
Right Femoral Neck BMD	−0.217	.004*	0.075	.326	0.181	.017*	0.302	<.001*
Right Hip BMD	−0.192	.011*	0.059	.439	0.179	.018*	0.236	.002*
Left Femoral Neck BMD	−0.246	.001*	0.062	.415	0.178	.019*	0.233	.002*
Left Hip BMD	−0.189	.013*	0.119	.120	0.225	.003*	0.280	<.001*
Total BMD	−0.238	.002*	0.111	.147	0.215	.005*	0.295	<.001*

* *p* ≤ .05 is considered statistically significant.

Correlation is seen using spearman’s rho correlation coefficient.

In a multivariate linear stepwise regression analysis, serum levels of chemerin, osteoprotegerin, vaspin, and omentin-1 were used to predict BMD at the lumbar spine, right femoral neck, right hip, left femoral neck, left hip, and total BMD. At the lumbar spine, the model accounted for approximately 8% of BMD variance (*R* ^2^ = 0.088, Adjusted *R* ^2^ = 0.077). The independent predictors were serum osteoprotegerin and serum chemerin levels, which accounted for 7% and 2% of the variance in T-scores at the lumbar spine, respectively (Table 3). The model explained about 10% of BMD variance at the right femoral neck (R2 = 0.106, Adjusted R2 = 0.096). Again, serum osteoprotegerin and serum chemerin levels were independent predictors, accounting for 9% and 3% of the variance in T-scores, respectively. The model accounted for about 5% of the variance in BMD at the right hip (R2 = 0.053, Adjusted R2 = 0.048). Serum osteoprotegerin levels were the only independent predictor. Similarly, at the left femoral neck, the model accounted for approximately 7% of BMD variance (*R* ^2^ = 0.076, Adjusted *R* ^2^ = 0.065). The independent predictors were serum osteoprotegerin and serum chemerin levels, which accounted for 5% and 4% of the variance in T-scores, respectively.

**TABLE 3:** Multivariate linear stepwise regression analysis showing independent predictors of BMD at various sites.

	Model		*R* ^2^	Adj *R* ^2^	Unstandardized coefficients		Standardized coefficients	t	Sig
					B	Std. Error	Beta		
BMD at lumbar spine	1	(Constant)	0.066	0.061	−3.570	0.564		−6.330	0.000
		Osteoprotegerin			0.156	0.045	0.257	3.484	0.001
	2	(Constant)	0.088	0.077	−3.562	0.559		−6.371	0.000
		Osteoprotegerin			0.167	0.045	0.274	3.723	0.000
		Chemerin			−0.306	0.152	−0.148	−2.008	0.046
BMD at right femoral neck	1	(Constant)	0.077	0.072	−2.979	0.497		−6.000	0.000
		Osteoprotegerin			0.149	0.039	0.278	3.782	0.000
	2	(Constant)	0.106	0.096	−2.970	0.490		−6.061	0.000
		Osteoprotegerin			0.160	0.039	0.298	4.077	0.000
		Chemerin			−0.314	0.133	−0.172	−2.355	0.020
BMD at right hip	1	(Constant)	0.053	0.048	−2.290	0.461		−4,966	0.000
		Osteoprotegerin			0.114	0.037	0.231	3.101	0.002
BMD at left femoral neck	1	(Constant)	0.040	0.034	−2.373	0.480		−4.948	0.000
		Osteoprotegerin			0.102	0.038	0.200	2.671	0.008
	2	(Constant)	0.076	0.065	−2.364	0.472		−5.008	0.000
		Osteoprotegerin			0.113	0.038	0.222	2.990	0.003
		Chemerin			−0.328	0.129	−0.190	−2.554	0.012
BMD at left Hip	1	(Constant)	0.074	0.068	−2.361	0.452		−5.226	0.000
		Osteoprotegerin			0.132	0.036	0.271	3.686	0.000
	2	(Constant)	0.102	0.091	−2.405	0.447		−5.384	0.000
		Osteoprotegerin			0.120	0.036	0.246	3.346	0.001
		Omentin-1			0.019	0.008	0.170	2.316	0.022
Total BMD	1	(Constant)	0.074	0.069	−2.715	0.445		−6.103	0.000
		Osteoprotegerin			0.131	0.035	0.272	3.695	0.000
	2	(Constant)	0.104	0.094	−2.707	0.439		−6.168	0.000
		Osteoprotegerin			0.140	0.035	0.292	3.997	0.000
		Chemerin			−0.286	0.119	−0.175	−2.392	0.018

Dependent variable is BMD.

Independent variables (predictors) used in the model were serum chemerin, osteoprotegerin, vaspin and omentin-1.

*p*-value ≤ .05 is considered statistically significant.

The model explained approximately 15% of BMD variance at the left hip (R2 = 0.147, Adjusted R2 = 0.126). The independent predictors were serum osteoprotegerin and omentin-1 levels that accounted for 7% and 5% of the variance of T-scores, respectively. For total BMD, the model accounted for approximately 10% of the variance in BMD (*R* ^2^ = 0.104, Adjusted *R* ^2^ = 0.094). The independent predictors were serum osteoprotegerin and serum chemerin levels, which accounted for 8% and 3% of the variance in T-scores, respectively (Table 3).

**FIGURE 2:**
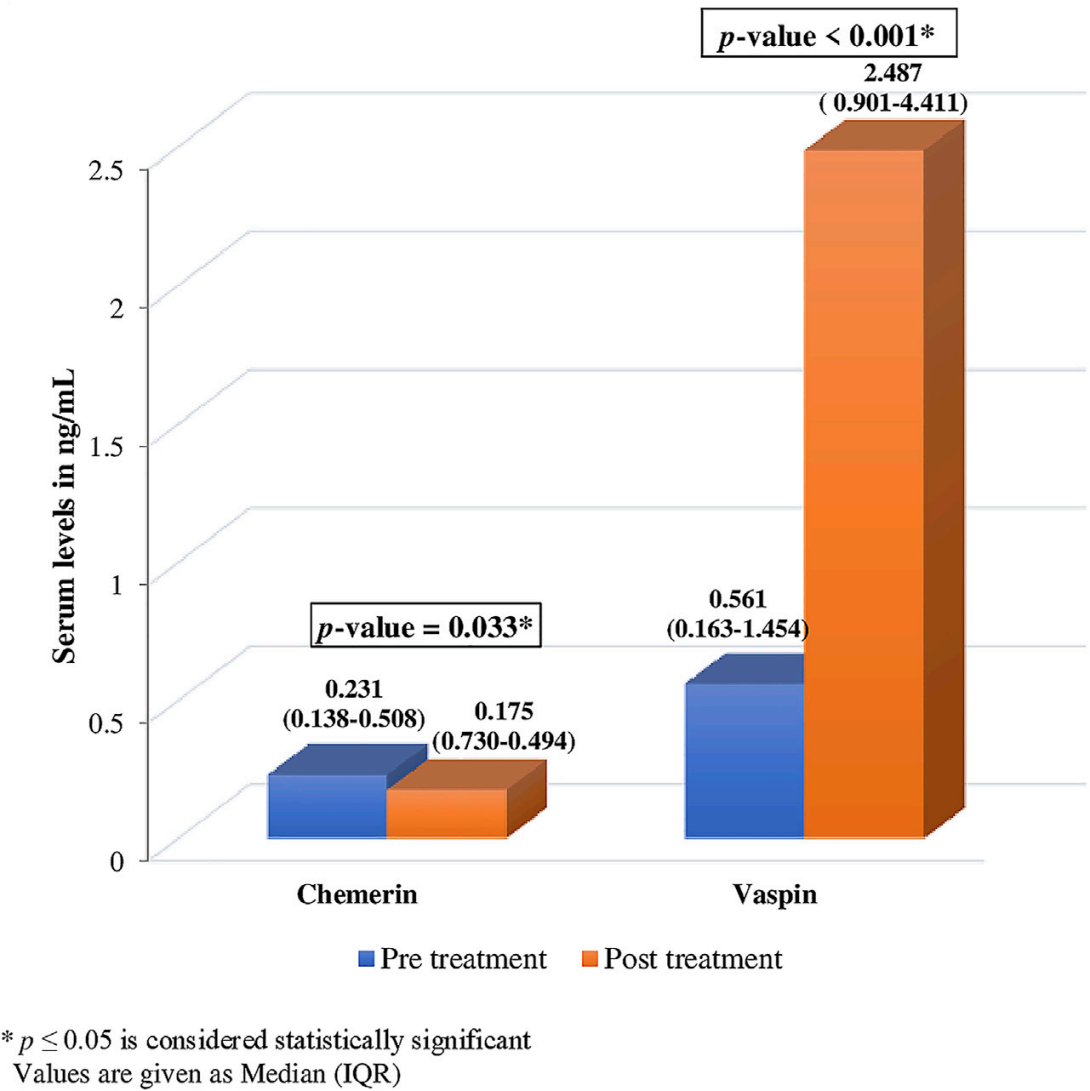
Comparison of pre and post treatment levels of serum chemerin and vaspin.

Serum chemerin and vaspin levels changed significantly after ibandronate treatment [chemerin 0.231 (0.138-0.508) ng/ml vs. 0.175 (0.730-0.494) ng/ml; vaspin 0.561 (0.163-1.454) ng/ml vs. 2.487 (0.901-4.411) ng/ml]. After treatment, chemerin levels decreased by 24.24% (*p* = .033*), while vaspin levels increased by 343.32% (p .001*) (Figure 2).

**FIGURE 3:**
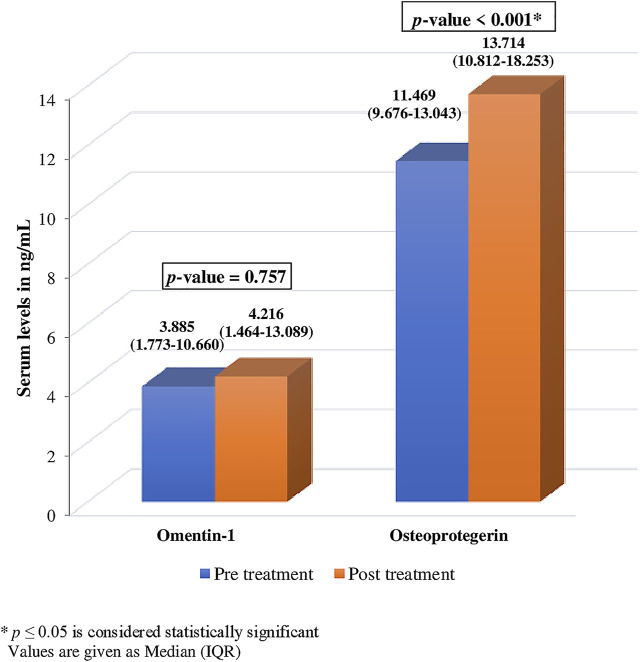
Comparison of pre and post treatment levels of serum omentin-1 and osteoprotegerin.

There was no significant change observed in the levels of serum omentin-1 after treatment with ibandronate (*p* = .757). However, a notable change was observed in the levels of serum OPG after treatment (Figure 3). The level of OPG changed significantly after ibandronate [11.469 (9.676-13.043) ng/ml, vs. 13.714 (10.812-18.253) ng/ml]. This depicts a 19.57% increase in OPG levels following treatment (p 0.001*).

## Discussion

This study found a significant relationship between various adipokines and BMD. Serum chemerin had a significant negative correlation with BMD, indicating that BMD increases with a decrease in serum chemerin levels. The current study’s findings on the relationship between chemerin and BMD were consistent with several other studies that found a negative correlation between chemerin and BMD in either osteoporotic or non-osteoporotic subjects (Terzoudis et al., 2016; Rana Ali Hamdi, 2018). In contrast to the current study’s findings, a study found no relationship between BMD and serum chemerin in lean and overweight subjects (Kadric et al., 2018), while another study noted a positive correlation in postmenopausal osteoporotic patients (Engin-Üstün et al., 2016). The strength of this negative association became prominent with the regression analysis, where chemerin was the predictor of BMD levels at various sites.

These differences could be due to chemerin’s ability to increase the function of both osteoblasts and osteoclasts (Neumann et al., 2016). Chemerin-induced bone erosion is facilitated by the fact that knocking out chemerin or its receptors in bone marrow stromal cells increased the expression of osteoblastic genes, indicating that chemerin has a negative effect on bone formation (Muruganandan et al., 2010). In the case of bone formation, chemerin appears to advance bone development. Chemerin might have a paracrine and autocrine role in bone homeostasis, and it is neutralization by chemerin that reduces osteoclast differentiation (Muruganandan et al., 2013; Zhao et al., 2019). The net effect would be an override or neutralization, depending on whichever drive was stronger, resulting in a negative, positive, or no association with BMD.

In the current study, chemerin levels were also reduced by 24.24% after treatment with Ibandronate (*p* = .033*). This is the first study exploring the levels of chemerin after bisphosphonate therapy. Our results could not be corroborated, as no comparable clinical study on these parameters was available. However, because chemerin levels decreased after bisphosphonate therapy, it is clear that ibandronate protects bones from chemerin-induced bone erosion by lowering chemerin levels. In a study of rheumatoid arthritis patients, serum chemerin levels were measured before and after 6 months of treatment with tocilizumab, and it was discovered that serum chemerin levels were significantly reduced after treatment with interleukin-6 receptor inhibitor, indicating that increased chemerin levels may result in bone pathology and that decreasing chemerin levels with treatment resulted in clinical response in such patients (Makrilakis et al., 2015).

A significant positive association was also found between serum vaspin and lumbar spine BMD, but not with BMD at other sites. There was a significant change in vaspin levels after ibandronate treatment (*p* < .001*). A study found that vaspin reduces the process of osteoclastogenesis induced by RANKL in the RAW264.7 cell line, resulting in increased bone mass (Kamio et al., 2013). Furthermore, it was discovered that vaspin could inhibit osteoblast programmed cell death via the extracellular signal-regulated kinase pathway (Zhu et al., 2013). Ibandronate’s increase in serum vaspin levels may be one of the mechanisms by which it increases BMD. Vaspin reduced H_2_O_2_-induced apoptosis in mice by inhibiting mesenchymal stem cell apoptosis, which is responsible for osteoporosis development, demonstrating its ability to protect against osteoporosis (Zhu et al., 2019). A study demonstrated no association of serum vaspin in patients taking bisphosphonate therapy and treatment naïve patients. However, the difference in their and our study was that they measured vaspin levels once in all patients and did not follow up the patients as we did in our study (Tanna et al., 2017).

Serum omentin-1 was found to have a significant positive relationship with BMD in postmenopausal females and it was also a strong predictor of BMD at the left hip. Although no significant change was observed after treatment with ibandronate. Many studies have witnessed a similar positive association between serum omentin-1 and BMD. A study reported that Omentin-1 stimulated human osteoblastic cells in a dose-dependent manner. When cells were treated with omentin-1 at concentrations of 25, 50, 100, and 200 ng/ml compared to the control group, there was a significant increase in osteoblastic cells proliferation (*p* < .05). Further investigation revealed that it is the Protein kinase B (PKB), also known as Akt, a pathway involved in the proliferation of osteoblastic cells through omentin-1 (Wu et al., 2013). Other *in vitro* and *in vivo* studies have revealed that omentin-1 protects BMD and the bone remodeling process (Kanaya and Chen, 2010; Xie et al., 2012). A study reported found a positive association between omentin-1 and OPG (Menzel et al., 2016). However, no such association between omentin-1 and OPG was discovered in the current study. Omentin-1 is also thought to protect against osteoporosis by inhibiting pro-inflammatory cytokines (Rao et al., 2018).

In contrast to our study, many studies found an inverse relationship between omentin-1 and BMD (Tohidi et al., 2012). It has also been reported that an increase in omentin-1 in osteoporosis and this inverse relationship with BMD could be due to a physiologic compensation for bone loss after menopause (Dikker et al., 2018).

**FIGURE 4:**
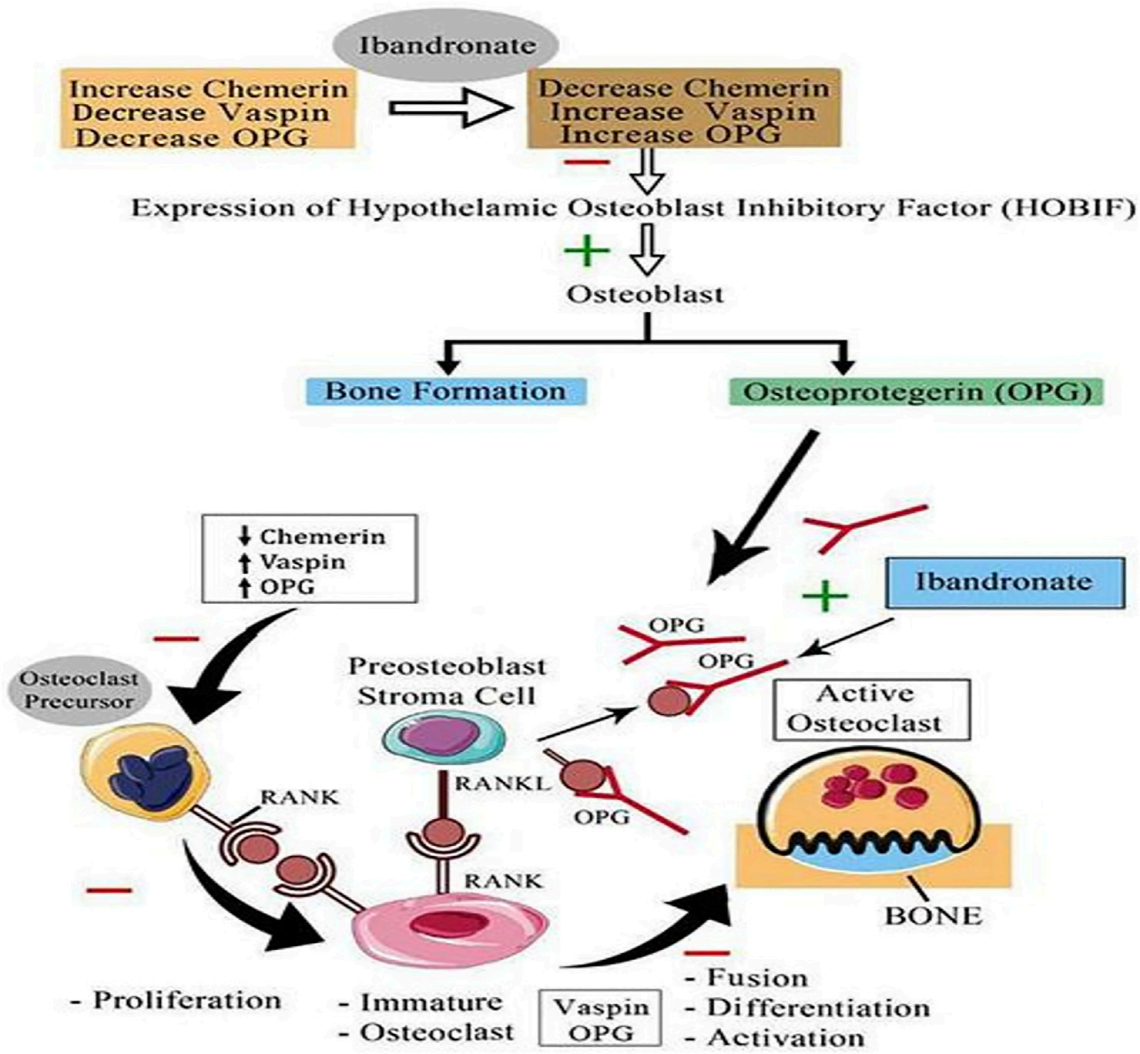
Proposed mechanism of action of bisphosphonate (Ibandronate) on serum chemerin, vaspin and OPG; HOBIF, hypothalamic osteoblast inhibitory factor; OPG, osteoprotegerin; RANK, receptor activator of nuclear factor kappa-B ligand; RANK L, receptor activator of nuclear factor kappa-B ligand or osteoprotegerin ligand; inhibit; −, stimulate +; ↑, increase; ↓ decrease.

The current study noticed a significant change in OPG levels following ibandronate treatment and a significant positive association of OPG with BMD at various sites; additionally, the strength of the positive association became more apparent with the regression analysis. However, there was no significant correlation between serum OPG and chemerin, vaspin, or omentin-1. It has been reported that RANKL causes activation of osteoclast, and OPG blocks this activation and suppresses the action of osteoclast, which are the main cells responsible for bone resorption (Gorecki et al., 2015). Based on our findings, we propose a possible mechanism through which ibandronate affects these adipokines and prevents osteoporosis (Figure 4).

Another study looked at the effect of bisphosphonate on OPG mRNA and human osteoblastic cell production at the molecular level and found that bisphosphonate increases OPG gene expression and increases protein secretion in human osteoblasts in a dose-dependent manner, which is consistent with our findings. They also observed a 6-fold increase in the effects of bisphosphonates on osteoblastic OPG protein secretion over time (Koch et al., 2012).

In contrast to our findings, a study conducted on postmenopausal females found that administration of raloxifene decreases the levels of OPG in these females. However, they demonstrated the raloxifene effect, which had a different chemical structure than ibandronate, and their sample size was also small (Bashir et al., 2005). Contrary to our findings, there was no change in serum OPG levels in osteoporotic postmenopausal women treated with ibandronate and other bisphosphonates (Stuss et al., 2016; Passeri et al., 2019). We also found a positive association of serum OPG levels with BMD. Another study discovered a similar positive relationship between OPG and BMD (Pandey et al., 2018). According to a recent study, women with low BMD had lower median serum levels of OPG than women with normal BMD (Azizieh et al., 2019). Lower circulating OPG concentrations were also independently and positively correlated with low BMD in postmenopausal females (Mezquita-Raya et al., 2005).

It is also worth noting that bone density is affected by diverse factors. These effects have an impact on bone *via* a variety of complex pathways. As a result, the conclusive role of single or multiple variables necessitates extensive research. The role of adipokines such as chemerin, vaspin, and omentin-1 is not well established. More research is needed before these adipokines can be used as potential therapeutic agents to prevent or treat osteoporosis in postmenopausal women.

## Conclusion

Adipokines have a significant correlation with BMD. After 6 months of ibandronate treatment, there was a significant change in serum chemerin, vaspin, and OPG levels. It seems that higher serum chemerin and lower vaspin and OPG levels could be implicated in the development or progression of osteoporosis. There was no effect of ibandronate treatment on serum levels of omentin-1 in our study subjects.

## Limitations

Aside from these novel adipokines, bone biomarkers can also be measured. We only performed a DEXA Scan on the patients once during the study because the literature indicates that significant changes usually occur after 2 years. Furthermore, due to financial constraints, the serum levels of adipokines in the control group were checked once to ensure that they were within the normal range in our population.

## Recommendations

• An extended study with larger sample size and a longer duration could be conducted to investigate the long-term effects of bisphosphonate on serum adipokines and their correlation with BMD.
• Furthermore, a comparison of gender differences should also be explored by conducting the same study in the male population to determine bisphosphonates’ effects on these adipokines.

## Data Availability

The raw data supporting the conclusion of this article will be made available by the authors, without undue reservation.

## References

[B1] Azizieh F. Y. Shehab D. Jarallah K. A. Gupta R. Raghupathy R. (2019). Circulatory Levels of RANKL, OPG, and Oxidative Stress Markers in Postmenopausal Women with normal or Low Bone mineral Density. Biomark Insights 14, 1177271919843825. 10.1177/1177271919843825 31452599 PMC6700864

[B2] Bashir A. Mak Y. T. Sankaralingam S. Cheung J. McGowan N. W. Grigoriadis A. E. (2005). Changes in RANKL/OPG/RANK Gene Expression in Peripheral Mononuclear Cells Following Treatment with Estrogen or Raloxifene. Steroids 70, 847–855. 10.1016/j.steroids.2005.04.011 16005483

[B3] Dikker O. Bekpinar S. Ozdemirler G. Uysal M. Vardar M. Atar S. (2018). Evaluation of the Relation between Omentin-1 and Vitamin D in Postmenopausal Women with or without Osteoporosis. Exp. Clin. Endocrinol. Diabetes 126, 316–320. 10.1055/s-0043-120110 29117613

[B4] Engin-Üstün Y. Çağlayan E. K. Göçmen A. Y. Polat M. F. (2016). Postmenopausal Osteoporosis Is Associated with Serum Chemerin and Irisin but Not with Apolipoprotein M Levels. J. Menopausal Med. 22, 76–79. 10.6118/jmm.2016.22.2.76 27617241 PMC5016507

[B5] Falaschi P. Giordano S. (2017). “Osteoporosis in Elderly Patients,” in Orthogeriatrics (Cham: Springer), 31–45. 10.1007/978-3-319-43249-6_3

[B6] Fatima S. S. Rehman R. Baig M. Khan T. A. (2014). New Roles of the Multidimensional Adipokine: Chemerin. Peptides 62, 15–20. 10.1016/j.peptides.2014.09.019 25278490

[B7] Gorecki P. Stockmann P. Distler J. H. W. Wuest W. Schmidt D. Neukam F. W. (2015). Implication of Bisphosphonate Use in the Treatment of SAPHO Syndrome: Case Report and Discussion of Current Literature. J. Med. Hypotheses Ideas 9, 72–78. 10.1016/j.jmhi.2015.04.002

[B8] Hagino H. Ito M. Hashimoto J. Yamamoto M. Endo K. Katsumata K. (2018). Monthly Oral Ibandronate 100 Mg Is as Effective as Monthly Intravenous Ibandronate 1 Mg in Patients with Various Pathologies in the MOVEST Study. J. Bone Miner. Metab. 36 (3), 336–343. 10.1007/s00774-017-0839-2 28389932

[B9] Kadric L. Zylla S. Nauck M. Völzke H. Friedrich N. Hannemann A. (2018). Associations between Plasma Chemerin Concentrations and Bone Quality in Adults from the General Population. Endocrinology 159, 2378–2385. 10.1210/en.2018-00157 29701774

[B10] Kamio N. Kawato T. Tanabe N. Kitami S. Morita T. Ochiai K. (2013). Vaspin Attenuates RANKL-Induced Osteoclast Formation in RAW264.7 Cells. Connect. Tissue Res. 54, 147–152. 10.3109/03008207.2012.761978 23323745

[B11] Kanaya N. Chen S. (2010). Conjugated Linoleic Acid Reduces Body Weight Gain in Ovariectomized Female C57BL/6J Mice. Nutr. Res. 30, 714–721. 10.1016/j.nutres.2010.09.001 21056287 PMC3000560

[B12] Koch F. P. Merkel C. Ziebart T. Smeets R. Walter C. Al-Nawas B. (2012). Influence of Bisphosphonates on the Osteoblast RANKL and OPG Gene Expression *In Vitro* . Clin. Oral Investig. 16, 79–86. 10.1007/s00784-010-0477-8 20938793

[B13] Li N. X. Tu Y. Liu X. X. Shen Y. Zhang L. H. (2016). Changes of Inflammatory Factors and Adipokines in Patients with Diabetic Osteoporosis. J. Hainan Med. Univ. 22, 25. 10.12659/msm.895759

[B14] Liu Y. Song C. Y. Wu S. S. Liang Q. H. Yuan L. Q. Liao E. Y. (2013). Novel Adipokines and Bone Metabolism. Int. J. Endocrinol. 2013, 895045. 10.1155/2013/895045 23431296 PMC3575660

[B15] Makrilakis K. Fragiadaki K. Smith J. Sfikakis P. P. Kitas G. D. (2015). Interrelated Reduction of Chemerin and Plasminogen Activator Inhibitor-1 Serum Levels in Rheumatoid Arthritis after Interleukin-6 Receptor Blockade. Clin. Rheumatol. 34, 419–427. 10.1007/s10067-014-2704-1 24912961

[B16] Mann M. C. Exner D. V. Hemmelgarn B. R. Hanley D. A. Turin T. C. MacRae J. M. (2014). The VITAH Trial Vitamin D Supplementation and Cardiac Autonomic Tone in Hemodialysis: a Blinded, Randomized Controlled Trial. BMC Nephrol. 15, 129–9. 10.1186/1471-2369-15-129 25098377 PMC4130113

[B17] Martini G. Gennari L. Merlotti D. Salvadori S. Franci M. B. Campagna S. (2007). Serum OPG and RANKL Levels before and after Intravenous Bisphosphonate Treatment in Paget's Disease of Bone. Bone 40, 457–463. 10.1016/j.bone.2006.08.003 16979395

[B18] Menzel J. Di Giuseppe R. Biemann R. Aleksandrova K. Kuxhaus O. Wittenbecher C. (2016). Association between Omentin-1, Adiponectin and Bone Health under Consideration of Osteoprotegerin as Possible Mediator. J. Endocrinol. Invest. 39, 1347–1355. 10.1007/s40618-016-0544-3 27614458 PMC5069301

[B19] Mezquita-Raya P. De la Higuera M. García D. F. Alonso G. Ruiz-Requena M. E. De Dios Luna J. (2005). The Contribution of Serum Osteoprotegerin to Bone Mass and Vertebral Fractures in Postmenopausal Women. Osteoporos. Int. 16, 1368–1374. 10.1007/s00198-005-1844-1 15711777

[B20] Muruganandan S. Dranse H. J. Rourke J. L. McMullen N. M. Sinal C. J. (2013). Chemerin Neutralization Blocks Hematopoietic Stem Cell Osteoclastogenesis. Stem Cells 31, 2172–2182. 10.1002/stem.1450 23766088

[B21] Muruganandan S. Roman A. A. Sinal C. J. (2010). Role of chemerin/CMKLR1 Signaling in Adipogenesis and Osteoblastogenesis of Bone Marrow Stem Cells. J. Bone Miner Res. 25, 222–234. 10.1359/jbmr.091106 19929432

[B22] Neumann E. Junker S. Schett G. Frommer K. Müller-Ladner U. (2016). Adipokines in Bone Disease. Nat. Rev. Rheumatol. 12, 296–302. 10.1038/nrrheum.2016.49 27080691

[B23] Pandey A. Khan Y. A. Kushwaha S. S. Mohammed F. Verma A. (2018). Role of Serum Osteoprotegerin as a Diagnostic Indicator of Primary Osteoporosis in Perimenopausal and Postmenopausal Women: An Indian Perspective. Malays Orthop. J. 12, 31–35. 10.5704/MOJ.1803.006 PMC5920256 29725510

[B24] Park J. H. Lee N. K. Lee S. Y. (2017). Current Understanding of RANK Signaling in Osteoclast Differentiation and Maturation. Mol. Cell 40, 706–713. 10.14348/molcells.2017.0225 PMC5682248 29047262

[B25] Passeri E. Mazzaccaro D. Sansoni V. Perego S. Nano G. Verdelli C. (2019). Effects of 12-months Treatment with Zoledronate or Teriparatide on Intima-media Thickness of Carotid Artery in Women with Postmenopausal Osteoporosis: A Pilot Study. Int. J. Immunopathol Pharmacol. 33, 2058738418822439. 10.1177/2058738418822439 30791743 PMC6327329

[B26] Ramos-Junior E. S. Leite G. A. Carmo-Silva cc. Taira T. M. Neves K. B. Colón d. F. (2017). Adipokine Chemerin Bridges Metabolic Dyslipidemia and Alveolar Bone Loss in Mice. J. Bone Miner Res. 32, 974–984. 10.1002/jbmr.3072 28029186

[B27] Rana Ali Hamdi R. A. (2018). Measurement of Serum Chemerin and Deoxypyridinoline Levels in Iraqi Osteoporotic Postmenopausal Women with and without Metabolic Syn-Drome. ijrps 10, 1273–1278. 10.26452/ijrps.v10i2.420

[B28] Rao S. S. Hu Y. Xie P. L. Cao J. Wang Z. X. Liu J. H. (2018). Omentin-1 Prevents Inflammation-Induced Osteoporosis by Downregulating the Pro-inflammatory Cytokines. Bone Res. 6, 9–2. 10.1038/s41413-018-0012-0 29619269 PMC5876344

[B29] Rosen H. N. Rosen C. J. Schmader K. E. Mulder J. E. (2017). Bisphosphonate Therapy for the Treatment of Osteoporosis. Avaliable At: https://www.uptodate.com/contents/bisphosphonate-therapy-for-the-treatment-of-osteoporosis (Accessed Apr 15, 2021).

[B30] Stuss M. Sewerynek E. Król I. Stępień-Kłos W. Jędrzejczyk S. (2016). Assessment of OPG, RANKL, Bone Turnover Markers Serum Levels and BMD after Treatment with Strontium Ranelate and Ibandronate in Patients with Postmenopausal Osteoporosis. Endokrynol Pol. 67, 174–184. 10.5603/EP.a2016.0014 26884284

[B31] Tanna N. Patel K. Moore A. E. Dulnoan D. Edwards S. Hampson G. (2017). The Relationship between Circulating Adiponectin, Leptin and Vaspin with Bone mineral Density (BMD), Arterial Calcification and Stiffness: a Cross-Sectional Study in post-menopausal Women. J. Endocrinol. Invest. 40, 1345–1353. 10.1007/s40618-017-0711-1 28646476

[B32] Tariq S. Baig M. Shahzad M. (2017). Calcaneal Ultrasound Assessment of Bone Health and Association of Socio-Demographic Characteristics with Bone mineral Density in Pre and Postmenopausal Females. Osteoporos. Int 28, S173–S174. 10.12669/pjms.35.3.551

[B33] Terzoudis S. Malliaraki N. Damilakis J. Dimitriadou D. A. Zavos C. Koutroubakis I. E. (2016). Chemerin, Visfatin, and Vaspin Serum Levels in Relation to Bone mineral Density in Patients with Inflammatory Bowel Disease. Eur. J. Gastroenterol. Hepatol. 28, 814–819. 10.1097/MEG.0000000000000617 26934527

[B34] Tohidi M. Akbarzadeh S. Larijani B. Kalantarhormozi M. Ostovar A. Assadi M. (2012). Omentin-1, Visfatin and Adiponectin Levels in Relation to Bone mineral Density in Iranian Postmenopausal Women. Bone 51, 876–881. 10.1016/j.bone.2012.08.117 22971441

[B35] Uehara M. Nakamura Y. Suzuki T. Nakano M. Takahashi J. (2021). Efficacy and Safety of Oral Ibandronate versus Intravenous Zoledronic Acid on Bone Metabolism and Bone Mineral Density in Postmenopausal Japanese Women with Osteoporosis. Jcm 10 (22), 5420. 10.3390/jcm10225420 34830702 PMC8624848

[B36] Watanabe T. Watanabe-Kominato K. Takahashi Y. Kojima M. Watanabe R. (2017). Adipose Tissue-Derived Omentin-1 Function and Regulation. Compr. Physiol. 7, 765–781. 10.1002/cphy.c160043 28640441

[B37] Weiner J. Zieger K. Pippel J. Heiker J. T. (2018). Molecular Mechanisms of Vaspin Action - from Adipose Tissue to Skin and Bone, from Blood Vessels to the Brain. Adv. Exp. Med. Biol., 1111, 159–188. 10.1007/5584_2018_241 30051323

[B38] Wu S. S. Liang Q. H. Liu Y. Cui R. R. Yuan L. Q. Liao E. Y. (2013). Omentin-1 Stimulates Human Osteoblast Proliferation through PI3K/Akt Signal Pathway. Int. J. Endocrinol. 2013, 368970. 10.1155/2013/368970 23606838 PMC3626246

[B39] Xie H. Xie P. L. Luo X. H. Wu X. P. Zhou H. D. Tang S. Y. (2012). Omentin-1 Exerts Bone-Sparing Effect in Ovariectomized Mice. Osteoporos. Int. 23, 1425–1436. 10.1007/s00198-011-1697-8 21755404

[B40] Zhao H. Yan D. Xiang L. Huang C. Li J. Yu X. (2019). Chemokine-like Receptor 1 Deficiency Leads to Lower Bone Mass in Male Mice. Cell Mol Life Sci 76, 355–367. 10.1007/s00018-018-2944-3 30374519 PMC11105338

[B41] Zhu X. Jiang Y. Shan P. F. Shen J. Liang Q. H. Cui R. R. (2013). Vaspin Attenuates the Apoptosis of Human Osteoblasts through ERK Signaling Pathway. Amino Acids 44, 961–968. 10.1007/s00726-012-1425-5 23135225

[B42] Zhu X. Zhang L. Chen Y. Chen B. Huang H. Lv J. (2019). Vaspin Protects Mouse Mesenchymal Stem Cells from Oxidative Stress-Induced Apoptosis through the MAPK/p38 Pathway. Mol. Cel Biochem 462, 107–114. 10.1007/s11010-019-03614-8 31463780

[B43] Zojer N. Brenner K. Beke D. Kudlacek S. Hawa G. Woloszczuk W. (2005). Bisphosphonate Treatment Does Not Affect Serum Levels of Osteoprotegerin and RANKL in Hypercalcemic Cancer Patients. Anticancer Res. 25 (5), 3607–3612. 16101188

